# Locally advanced rectal cancer in a young adult affected with dyskeratosis congenita (Zinsser–Cole–Engman syndrome): a case report

**DOI:** 10.1186/s40792-024-01985-9

**Published:** 2024-09-06

**Authors:** Kosuke Ono, Yukiharu Hiyoshi, Asuka Ono, Mayuko Ouchi, Keisuke Kosumi, Kojiro Eto, Satoshi Ida, Masaaki Iwatsuki, Yoshifumi Baba, Yuji Miyamoto, Ikko Kajihara, Kazuhito Tanaka, Yuko Miyasato, Hideo Baba

**Affiliations:** 1 Department of Gastroenterological Surgery, Graduate School of Medical Sciences, Kumamoto University, 1-1-1 Honjo, Kumamoto, 860-8556 Japan; 2 Department of Dermatology, Kumamoto University Hospital, Kumamoto, Japan; 3 Department of Diagnostic Pathology, Kumamoto University Hospital, Kumamoto, Japan

**Keywords:** Dyskeratosis congenita, Zinsser–Cole–Engman syndrome, Rectal cancer, Total neoadjuvant therapy, Non-operative management, Watch and wait, Total pelvic exenteration

## Abstract

**Background:**

Dyskeratosis congenita (DKC), also known as Zinsser–Cole–Engman syndrome, is a progressive genetic disease with a triad of reticulate skin pigmentation, nail dystrophy, and leukoplakia. Approximately 8–10% of patients with DKC develop malignancies, and cases of colorectal cancer with DKC in young people have been reported previously.

**Case presentation:**

A 25-year-old man with DKC since approximately 10 years of age developed fever and lower abdominal discomfort. Diagnostic imaging revealed locally advanced rectal cancer with lymph node metastasis, direct invasion of the prostate, and pelvic abscess due to tumor microperforation (cT4bN2M0 cStage IIIC). Biopsy showed well to moderately differentiated ductal adenocarcinoma. Genetic testing was negative for RAS and BRAF gene mutations, and microsatellite instability (MSI) testing was also negative. After sigmoid colostomy, the patient was treated with total neoadjuvant therapy (TNT) with systemic chemotherapy (six courses of FOLFOX + panitumumab) followed by chemoradiation therapy (50.4 Gy with capecitabine). After TNT, the primary tumor and metastatic lymph nodes shrank. According to the findings of colonoscopy and magnetic resonance image (MRI), we diagnosed near complete response (near-CR) and decided to follow the patient without surgery by every 3 months re-evaluation. However, 5 months after TNT, tumor regrowth was detected on colonoscopy and imaging, and the patient underwent total pelvic exenteration. He developed paralytic ileus as a postoperative complication, and was discharged on the 38th postoperative day. Pathological examination revealed a residual tumor with invasion of the periprostatic tissue. There was no metastasis in the pararectal and lateral pelvic lymph nodes, but one extramural non-contiguous cancerous extension (tumor deposit) was observed (ypT4bN1cM0 ypStage IIIC). The patient has been free of recurrence for one year after surgery.

**Conclusions:**

DKC often develops into various tumors in the digestive system at an early age; therefore, appropriate surveillance may be required. In addition, considering that cancers in patients with DKC occur at a young age, fertility preservation and survivorship are also important, and adequate explanations and care should be provided to patients before and after treatment.

## Introduction

Colorectal cancer (CRC) ranks third in the number of new cancer cases worldwide, with approximately two million cases, and second in the number of deaths among all cancers [1]. The overall incidence of CRC has declined over the past few years, possibly due to colonoscopy screening in patients > 50 years of age [2]. In contrast, the incidence of CRC among patients younger than 50 years has increased at rates of 1.5 and 1.6% in males and females, respectively. In Japan, approximately one million people are diagnosed with cancer annually, 2% of whom are adolescents and young adults (AYAs) between 15 and 39 years of age [3]. Among individuals with AYA in Japan, CRC is the fourth most common cancer after breast, uterine, and thyroid cancers [4].

Dyskeratosis congenita (DKC), also known as the Zinsser–Cole–Engman syndrome, is a progressive genetic disease characterized by a triad of reticulate skin pigmentation, nail dystrophy, and leukoplakia. It may be a multisystem disease involving hematological, gastrointestinal, genitourinary, neurological, ophthalmic, pulmonary, and skeletal systems [5]. The annual incidence is less than one per million. The condition is progressive and genetic, but it is difficult to diagnose at an early stage because the clinical features develop slowly in early youth. It has been reported that telomere maintenance is abnormal in DKC, which may predispose individuals to the development of malignant tumors [6]. Approximately 8–10% of patients with DKC develop malignant tumors, and there have been reports of rectal cancer complications, as occurred in the present case [6–8]. We report our experience with a young adult with lower advanced rectal cancer complicated by DKC along with a review of the relevant literature.

## Case presentation

**Fig. 1:**
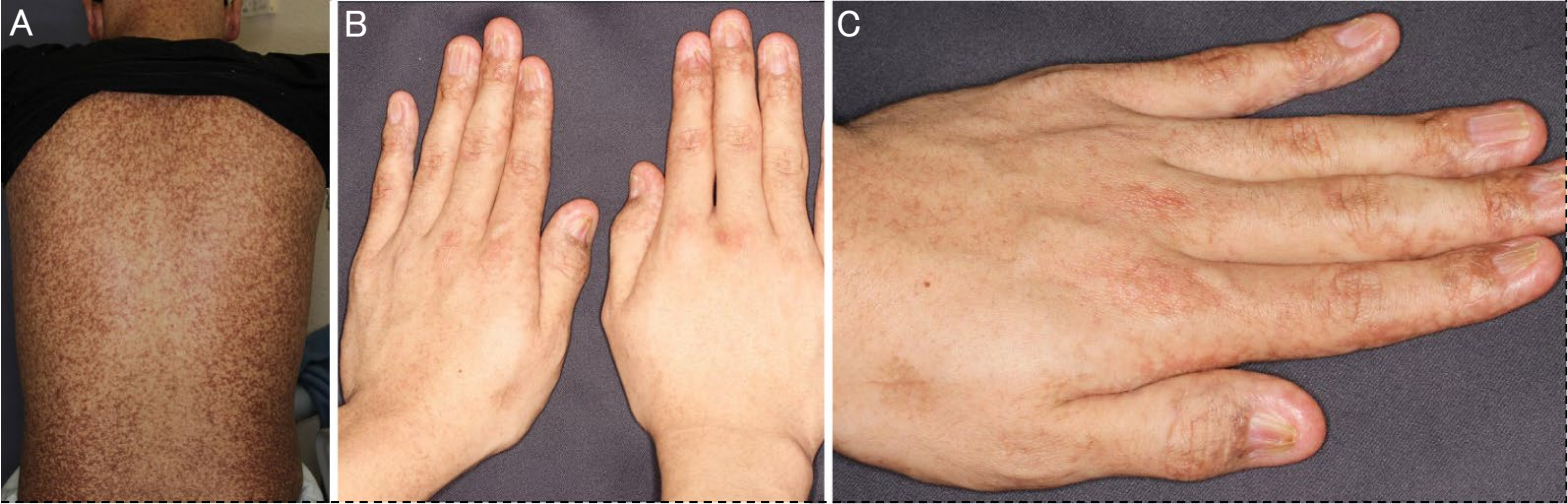
Images of skin findings. **A** Pigmentation is mainly observed on the skin of the trunk. **B**, **C** In addition to pigmentation of the dorsum of the hand, atrophic changes in the nail were noted

**Fig. 2:**
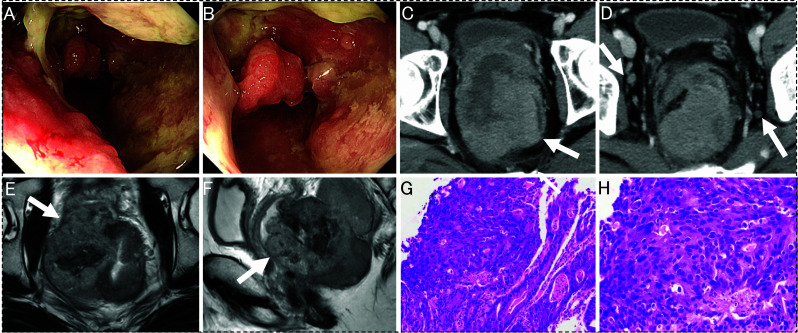
Images of rectal cancer before chemoradiotherapy. **A**, **B** Colonoscopy showing a near-circumferential type 2 tumor in the lower rectum. **C**, **D** Contrast-enhanced CT showing a massive lesion in the rectum (C; arrow). The tumor was widely attached to the right internal obturator muscle and direct invasion of the prostate was suspected. Swollen lymph nodes around the rectum and lateral pelvic area were detected (D; arrow). **E**, **F** MRI showing circumferential wall thickening in the rectum with a heterogeneous signal on T2-weighted images (E; arrow). Direct invasion of the prostate was suspected (F; arrow). **G**, **H** Biopsy revealed well to moderately differentiated ductal adenocarcinoma

A 25-year-old man was followed by a dermatologist for skin lesions (Fig. 1A–C) of DKC at approximately 10 years of age. The patient developed fever and lower abdominal discomfort. Computed tomography (CT) raised the suspicion of microperforation of the rectum due to advanced rectal cancer. The patient was treated with antibiotics, but the inflammation persisted, and a large tumor obstructed the rectal passage. Therefore, a laparoscopic colostomy was performed. After the surgery, the patient’s condition stabilized, and a close examination was performed. Colonoscopy revealed a 3/4 circumference lesion in the lower rectum (Fig. 2A, B). Imaging findings suggested invasion of the prostate and other surrounding organs, and pelvic lymph node metastasis was suspected (Fig. 2C–F), but no distant metastasis was found. In addition, the biopsy results showed well to moderately differentiated ductal adenocarcinoma (Fig. 2G–H). Genetic testing was negative for mutations in the RAS and BRAF genes, and microsatellite instability (MSI) testing was also negative. The tumor-node-metastasis (TNM) classification was cT4bN2M0, cStage IIIC.

**Fig. 3:**
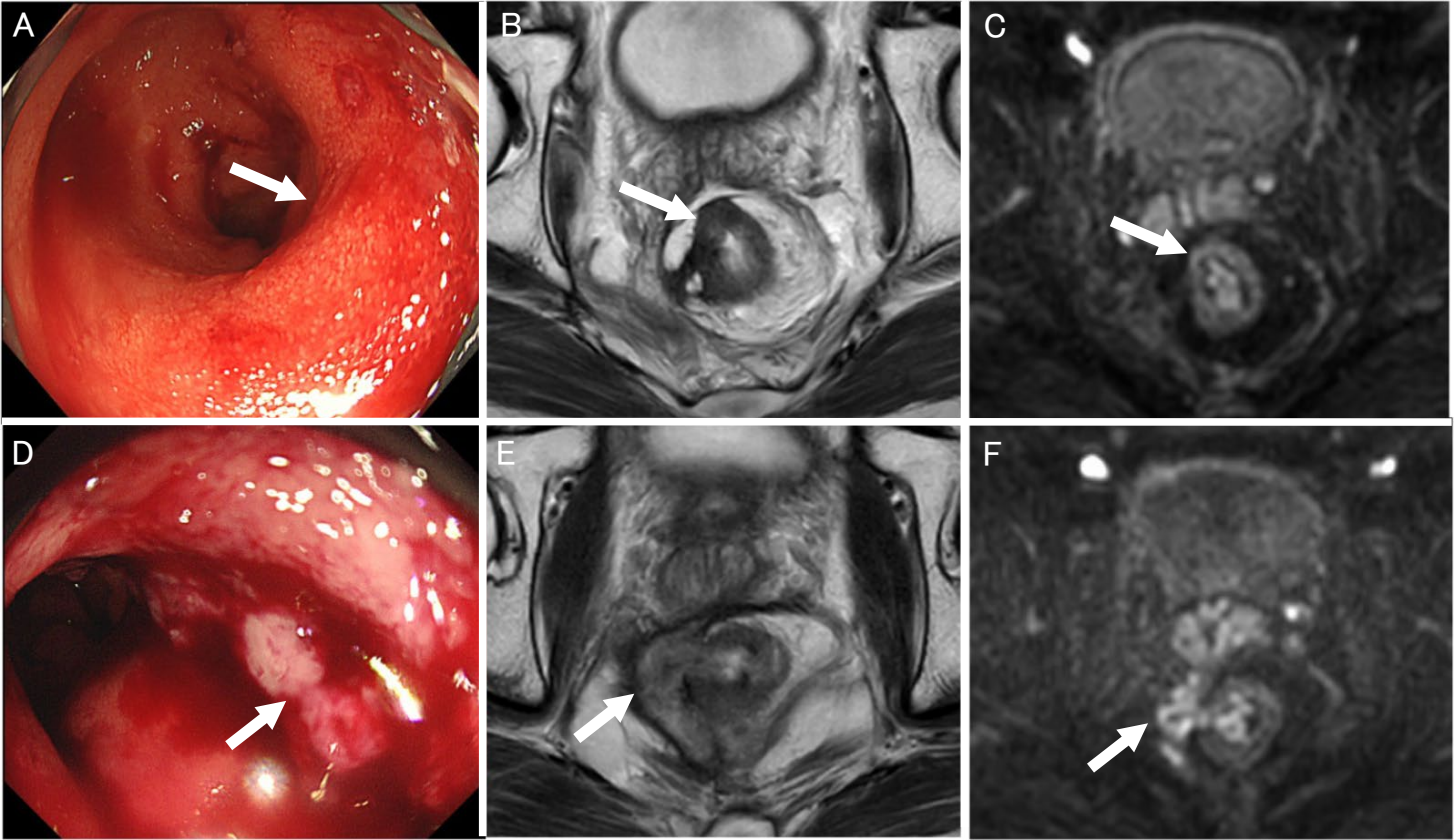
Images of rectal cancer after chemoradiotherapy and after regrowth. **A** Colonoscopy showed mucosal erythema (A; arrow) without residual tumor and a mildly narrowed rectum. **B**, **C** MRI showed mainly dark signal due to fibrosis and the lymph nodes with suspected metastasis had shrunk. However, some intermediate signals surrounded by dark signal on T2 (B; arrow), and slight diffusion restriction remained (C; arrow). The border of the prostate was preserved. **D** Tumor regrowth with an ulcer lesion detected on colonoscopy (D; arrow). **E**, **F** MRI showed abnormal signals extending from outside the rectal wall to the lumen (E; arrow). Direct invasion of the prostate was also suspected. Significant diffusion restriction was also observed in the same area (F; arrow). Regrowth of the tumor was suspected

After sigmoid colostomy, total neoadjuvant therapy (TNT) with induction therapy (6 courses of FOLFOX + panitumumab) was administered because multiple lymph node metastasis was suspected, in addition to prostate invasion. Accordingly, early systemic cancer control was considered necessary. Chemoradiation therapy (50.4 Gy with capecitabine) was then administered. The patients were reevaluated at 8 weeks after chemoradiotherapy. No treatment was given during that time. Eight weeks after TNT, a digital rectal examination did not reveal an obvious mass or nodule. Colonoscopy showed mucosal erythema (Fig. 3A) without residual tumor. Biopsy revealed acute inflammation with erosion or ulceration with without malignant findings. MRI showed mainly dark signal due to fibrosis and the lymph nodes with suspected metastasis had shrunk. However, some intermediate signals surrounded by dark signal on T2 and slight diffusion restriction remained (Fig. 3B, C). Based on these test results, the treatment effect was not reached clinical complete response (cCR) but judged as major response (near-CR). The patient was a young man who strongly desired to preserve organ function. The above assessments were performed 8 weeks after the end of CRT and considering that further efficacy could be expected after that, therefore, we decided to follow the patient without surgery by every 3 months re-evaluation. However, 3 months after first evaluation as near-CR, tumor regrowth was detected on colonoscopy and MRI (Fig. 3D–F). A tumor biopsy revealed a well to moderately differentiated ductal adenocarcinoma. We decided to perform a total pelvic exenteration, as we suspected direct invasion of the prostate.

During surgery, no obvious liver metastases or peritoneal dissemination was observed. The entire sacral area was difficult to dissect because the tumor volume prevented a clear view and fibrosis was strong. Therefore, a simultaneous perineal approach was also used. Postoperatively, the patient began to eat on day 9. Despite complications such as paralytic ileus, all drains were removed on the 16th postoperative day, and the patient was discharged on the 38th postoperative day.

**Fig. 4:**
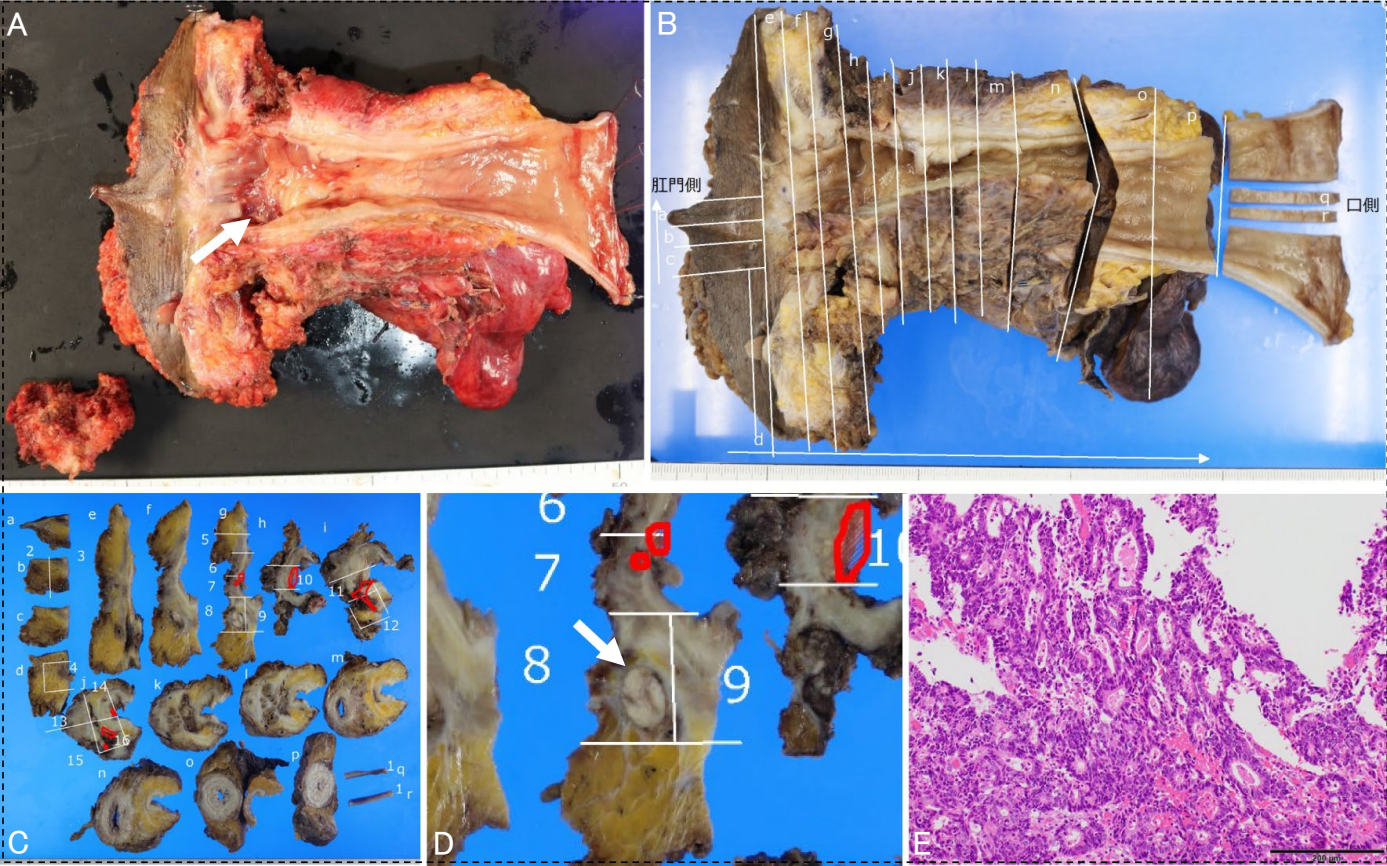
Image of resected specimen. **A** The size of the ulcer lesion in the rectum was approximately 30 × 20 mm (A; arrow). **B**–**E** A pathological examination revealed residual ductal adenocarcinoma, mainly of the moderately differentiated type (C; areas surrounded by red line). Although there was no obvious continuity with the main rectal tumor, cancerous invasion of the perineal prostatic tissue along with granulation tissue was observed. There was no metastasis in the pararectal and lateral pelvic lymph nodes, but one extramural non-contiguous cancerous extension (tumor deposit) (D; arrow) was observed in the pararectal lymph node. The TNM classification was ypT4bN1cM0 with ypStage IIIC

The postoperative pathology results showed a residual tumor, mainly moderately differentiated ductal adenocarcinoma (Fig. 4A–E). The tumor had crossed the intrinsic muscular layer and invaded the subserosa. There was cancerous invasion of the periprostatic tissues, along with granulation tissue. The resection margin was negative. The histological treatment effect of TNT was grade 1b. There was no metastasis in the pararectal and lateral pelvic lymph nodes, but one extramural non-contiguous cancerous extension (tumor deposit) was observed. The TNM classification was ypT4bN1cM0 with ypStage IIIC. Since systemic chemotherapy had been performed in the TNT strategy, the patient was observed without any postoperative adjuvant treatment. At the time of writing this report, one year has passed without recurrence.

## Discussion

DKC, originally reported by Zinsser in 1906, is a rare inherited bone marrow failure syndrome that is classically characterized by dysplastic nails, lacy reticular pigmentation of the upper chest and/or neck, and oral leukoplakia. Individuals with DKC often develop pulmonary fibrosis and malignancies. However, this phenotype is highly variable. The main causes of mortality are bone marrow failure and immune defects (60–70%), followed by pulmonary complications and cancer [7]. DKC is associated with defects in telomere biology. Patients with DKC have very short telomeres and mutations have been identified in genes associated with telomere biology. It is thought that abnormal telomere maintenance leads to the development of malignancies [6]. Approximately 8–10% of patients with DKC develop malignancies [6, 7]. The most frequent tumor type in patients with DKC is head and neck squamous cell carcinoma (40%), followed by skin and anorectal cancers. According to a previous report on colorectal cancer with DKC, the average age at the diagnosis of rectal cancer in six patients was 28 years, whereas two patients with colon cancer were diagnosed at 20 and 25 years of age, respectively [6]. In the present case, gastrointestinal screening was not performed during the course of DKC and advanced rectal cancer was detected when the patient was symptomatic. Although it is unknown how DKC is involved in cancer development, DKC often develops various tumors in the digestive system, including the rectum, stomach, esophagus, colon, pancreas, and liver, at an early age. Therefore, appropriate surveillance may be required, such as fecal occult blood testing, digital rectal examination, and/or endoscopic examination, from 10 years of age. Screening with CT may also be necessary for the liver and pancreas. Monthly self-examination for oral and head and neck cancer, annual cancer screening by an otolaryngologist, gynecological examination, and skin cancer screening by a dermatologist are recommended as surveillance for other malignancies [10]. In the case of rectal cancer, organ preservation and sexual function preservation at the time of surgery, and sperm and egg preservation (if radiotherapy or chemotherapy are required) are also considered. Fertility preservation and survivorship management through these measures should also be considered.

The incidence of AYA-CRC is up to 2000 patients per year, which accounts for approximately 10% of all cancers diagnosed in this age group [11]. A Japanese multicenter cohort study showed a slight male predominance (56.8%) in the incidence of AYA-CRC, with 54.4, 23.7, and 21.9% of tumors occurring in the rectum, left colon, and right colon, respectively [12]. The mortality rate of AYA-CRC has decreased in Japan, and the annual number of deaths due to CRC has decreased from 700 in 1975 to 250 in 2020. In the United States, on the other hand, the mortality rate of AYA-CRC is still increasing [13]. Although most cases of AYA-CRC are sporadic, approximately 30% of cases are thought to have a hereditary component [14]. While 3–5% of these have well-characterized hereditary cancer syndromes, such as Lynch syndrome, familial adenomatous polyposis (FAP), and other rare syndromes (e.g., *MutYH*-associated polyposis, Peutz–Jeghers syndrome, juvenile polyposis, polymerase proofreading-associated polyposis, and Cowden/PTEN hamartoma syndrome), other hereditary cases such as DKC remain unexplained [15]. Patients with this genetic background tend to develop CRC earlier, which is consistent with the present case. Pearlman et al*.* analyzed the prevalence of germline mutations associated with cancer susceptibility among 450 CRC patients younger than 50 years of age and revealed that 16% of patients had genetic cancer susceptibility [16]. Genetic testing should be considered for all AYAs with CRC, because important genetic abnormalities may be discovered in the wake of CRC. However, AYA-specific susceptibility has not been identified. For CRC occurring in the AYA generation, consideration should be given to performing DNA sequencing to rule out genetically related diseases. Early detection of genetically related diseases and early therapeutic intervention may be possible, for example, early detection of uterine cancer when Lynch syndrome is genetically suspected.

Advanced rectal cancers are traditionally treated with neoadjuvant chemoradiotherapy (nCRT) followed by total mesorectal excision (TME) and postoperative adjuvant chemotherapy [17, 18]. Surgery combined with radiotherapy was shown to significantly improve local control. However, it was not associated with improved overall survival, mainly due to distant metastasis after surgery [19]. Marginal or poor overall survival results from micrometastatic disease during the waiting period between nCRT and surgery and poor compliance with postoperative adjuvant chemotherapy. Recent evidence has shown that the achievement of pathologic complete response (pCR) after nCRT is associated with a good prognosis [20, 21]. Current rectal cancer management should be extended to allow a greater chance of achieving a pCR and an organ-preserving strategy should be applied. This strategy should be beyond sphincter-preserving surgery due to its many disadvantages (e.g., low anterior resection syndrome, sexual and voiding difficulties, and permanent colostomy) [21, 22]. Various approaches have been tested to improve the survival of patients with rectal cancer, and have resulted in promising outcomes. A new treatment strategy, total neoadjuvant therapy (TNT), consists of chemotherapy administered before or after CRT to control micrometastatic disease earlier and to improve compliance with chemotherapy. The majority of local recurrences during a watch-and-wait strategy are seen in the intestinal lumen within 12–18 months. Therefore, frequent rectal and endoscopic examinations during the first 2 years are the basis for local follow-up. Supplemental MRI is necessary to detect regrowth deep in the intestinal wall or into lymph nodes. The basic treatment for re-growth is surgical therapy with TME [23]. In addition, the number of clinical trials examining organ preservation strategies such as non-operative watch-and-wait management in patients with rectal cancer is progressively increasing [23]. In this case, the patient was diagnosed with advanced rectal cancer with suspected direct invasion of the prostate, and pelvic lymph node swelling was observed. Therefore, TNT was performed to control cancer both locally and systemically. Significant tumor shrinkage (near-CR) was achieved with TNT, and we decided to perform non-operative management for organ preservation. Unfortunately, total pelvic exenteration was required due to tumor regrowth with invasion of the prostate. Because this patient originally had prostate invasion, total pelvic exenteration was unavoidable because of tumor regrowth. Regrowth of the tumor located on the anterior wall requires caution, as it may invade the urinary tract system and require extended surgery.

In this report, we presented the case of locally advanced rectal cancer in a 27-year-old man with DKC, also known as the Zinsser–Cole–Engman syndrome. The rectal cancer was treated with TNT and a watch-and-wait strategy, followed by surgery after tumor regrowth. Appropriate surveillance may be required as patients with DKC often develop various tumors in the digestive system at an early age. In addition, considering that cancers in patients with DKC occur at a young age, fertility preservation and survivorship are also important, and adequate explanations and care should be provided to patients with AYA-CRC before and after treatment.

## Data Availability

All data generated or analyzed during this study are included in this published article.

## Abbreviations

DKC: Dyskeratosis congenita
MSI: Microsatellite instability
TNT: Total neoadjuvant therapy
cCR: Clinical complete response
CRC: Colorectal cancer
AYA: Adolescents and young adults
CT: Computed tomography
TNM: Tumor-node-metastasis
FOLFOX: Fluorouracil, levofolinate, oxaliplatin
Pmab: Panitumumab
FAP: Familial adenomatous polyposis
nCRT: Neoadjuvant chemoradiotherapy
TME: Total mesorectal excision
pCR: Pathologic complete response
